# Acute Eosinophilic Ascites in a Middle-Aged Man

**DOI:** 10.1155/2012/896523

**Published:** 2012-05-08

**Authors:** Fadi Bleibel, Keith Fragoza, Garrey T. Faller

**Affiliations:** ^1^Department of Internal Medicine, Steward Carney Hospital, Tufts University, Boston, MA 02124, USA; ^2^Department of Pathology, Steward Carney Hospital, Tufts University, Boston, MA 02124, USA

## Abstract

Eosinophilic gastroenteritis is a rare condition characterized by recurrent eosinophilic infiltration of portions of the GI tract and presenting with nonspecific GI symptoms in association with peripheral eosinophilia. Its etiology and pathogenesis remain unclear and its symptoms overlap with many GI and systemic diseases. Thus, both gastroenterologists and general internists need to be aware of this rare condition. We present a case of a 55-year-old male with diffuse abdominal pain and distention for two weeks. His physical examination was significant for moderate ascites. Initial work-up demonstrated severe peripheral blood eosinophilia, normal liver function tests, thickening of the stomach and small bowel wall, and elevated serum IgE. Upper endoscopy and extensive testing for malignancy and parasitic infections failed to establish a diagnosis. Ascitic fluid analysis showed significant eosinophilia. Further, a full-thickness jejunal showed marked eosinophilic infiltration of the serosa and muscularis propria. Subsequent treatment with oral prednisone resulted in normalization of laboratory and radiologic abnormalities in a few week period.

## 1. Introduction

Eosinophilic gastroenteritis is a rare disease of the gastrointestinal system characterized by recurrent eosinophilic infiltration of portions of the GI tract. This disorder leads to nonspecific GI symptoms that are usually associated with peripheral eosinophilia. The etiology and pathogenesis of EGE are unclear and its symptoms overlap with many GI and systemic diseases. Thus it is an easily missed condition that needs more awareness from the gastroenterologists and general internists.

## 2. Case Presentation

A 55-year-old Vietnamese male with no significant past medical history presents to a local community hospital emergency department because of abdominal pain and distention of two-week duration. The abdominal pain was generalized, described as cramping, present throughout the day, had no association with meals, and was getting progressively worse. The patient also reported nausea and vomiting clear material. He denied any recent fever, chills, night sweats, weight loss, change in bowel habits, sick contacts, and consumption of raw food. In addition, he denied any chest pain, shortness of breath, joint swelling, and skin rash. After immigration from Vietnam 11 years ago, his only travel outside the USA was back to his homeland one year prior to this illness. The patient was not taking any prescribed or over the counter medications or herbal compounds and denied any allergies. His physical examination showed no skin rash or jaundice, cardiopulmonary examination showed no abnormality, and the abdomen was moderately distended, with active bowel sounds, diffuse tenderness without rebound, and moderate ascites. There was no hepatomegaly or abdominal mass.

**Figure 1:**
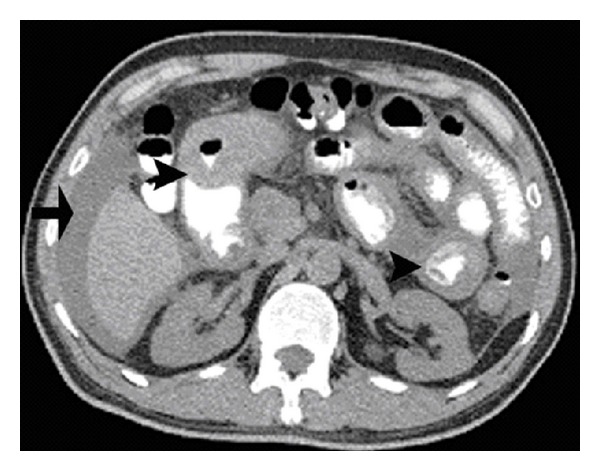
This computer tomography image of the abdomen and pelvis with oral and intravenous contrast shows multiple areas of small bowel wall thickening (arrowheads). Also notice the perihepatic accumulation of ascitic fluid (arrow).

In the emergency department, a complete blood count and comprehensive metabolic panel were significant for an elevated white blood count of 15.400 with 36% eosinophils. Abdominal and pelvis computer tomography (CT) showed moderate ascites with thickening of the gastric antrum and proximal small bowel (Figure 1). The patient was admitted to the general medical service and placed on bowel rest and intravenous fluid hydration. Further he underwent esophagogastroduodenoscopy (EGD), which demonstrated mild duodenitis and biopsies demonstrated mild nonspecific acute inflammation predominantly lymphocytic. Stool tests were negative for ova and parasites. In addition, a screen for Cryptosporidium, Cyclospora, Isospora, and Sarcocystis did not reveal evidence of recent infection. Over several days, the patient's abdominal pain improved, diet was advanced, and he was discharged home with an empiric trial of albendazole for a presumptive diagnosis of parasitic infection.

**Figure 2:**
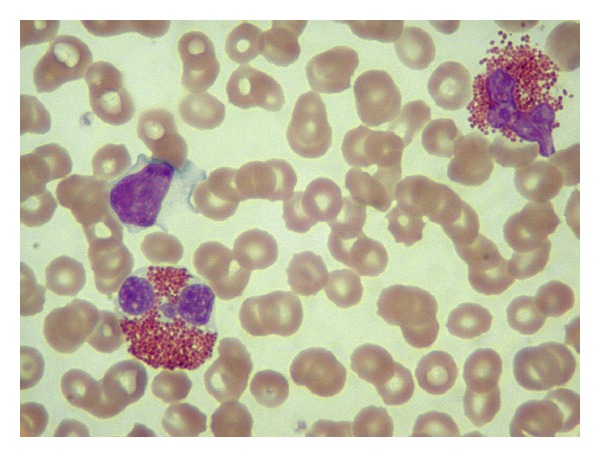
This high-power oil immersion view of the peripheral blood shows two eosinophils. In addition, a lymphocyte is present as well as abundant background of red blood cells (Wright-Giemsa stain; magnification 1000×).

Two weeks after discharge, the patient was readmitted with worsening abdominal pain. Physical examination showed increased abdomen distention. Repeated blood counts and serum biochemical tests demonstrated an increase in white cell count of 17.100/mL with 71% eosinophils (absolute eosinophil count of 12.141/*μ*l with normal upper limit <450) (Figure 2). Liver function tests continued to be within normal limits. Serum IgE level was elevated at 548 IU/mL (normal < 180). Repeat stool tests were negative for ova and parasites. Furthermore, immunologic studies for Toxocara, Trichinella, Strongyloides, Filiaria, Schistosoma, Echinococcus, and Cysticerus were negative.

**Figure 3:**
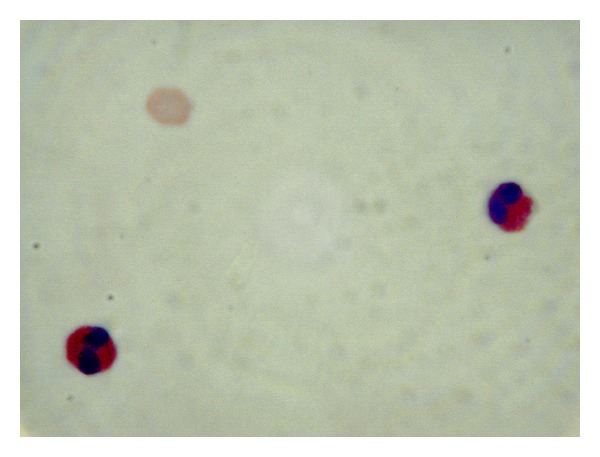
This high-power oil immersion view of ascitic fluid shows two degenerating eosinophils containing eosinophilic granules and bilobed nuclei (H&E stain; magnification 1000×).

**Figure 4:**
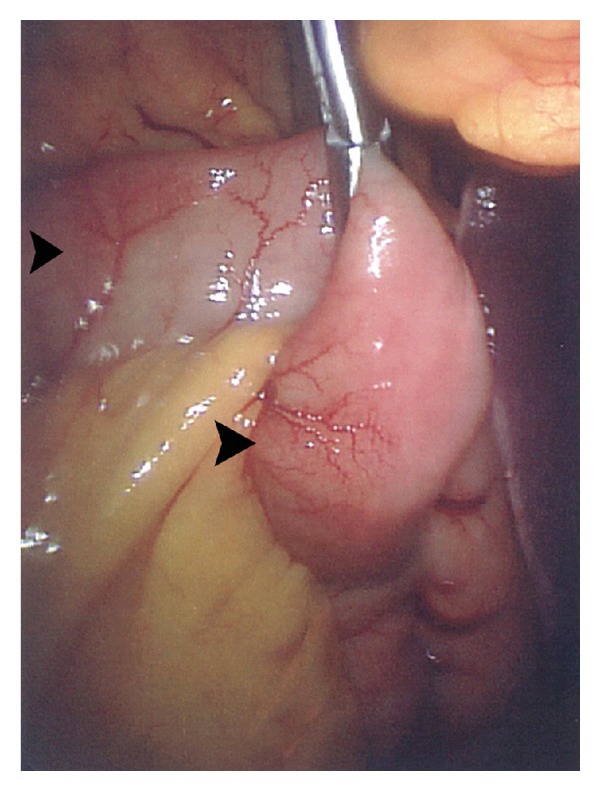
Laparoscopic evaluation of the patient revealed skipped areas of serosal inflammation involving the small bowel and to a lesser degree the colon. This view of the biopsied part of jejunum reveals an area of hyperemia and dilated blood vessels indicating an inflammatory process (arrowheads).

**Figure 5:**
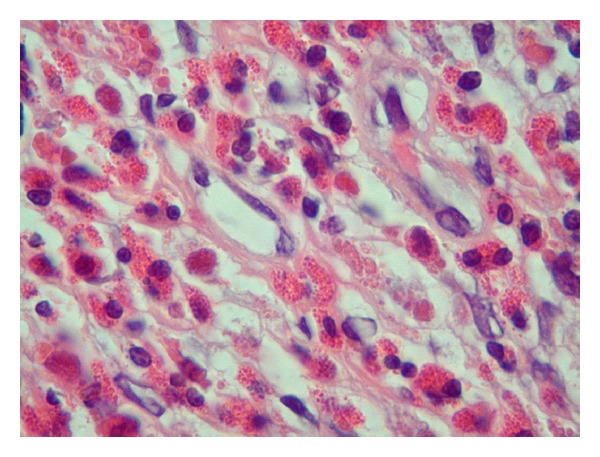
This high-power oil immersion view of the serosa shows sheets of eosinophils with abundant eosinophilic granules (H&E stain; magnification 1000×).

Repeated EGD was nondiagnostic. Flow cytometry of peripheral blood revealed no myelo- or lymphoproliferative findings. Serum *β*-2-microglobulin and LDH were 2.2 mg/dL (normal: 0.8–3.0) and 170 U/L (normal: 80–200), respectively. Ultrasound guided abdominal paracentesis showed WBC count of 6600/mL, 95% of which were eosinophils (Figure 3), LDH 284 mg/dL, albumin 3.2 g/dL (simultaneous serum albumin 4.1 g/dL). In order to exclude small bowel lymphoma, the patient underwent diagnostic laparoscopy with full-thickness biopsy of an inflamed portion of the jejunum. This revealed skipped areas of hyperemia and discoloration involving the small intestine and to a lesser degree the colon in addition to yellow-green ascites (Figure 4). Histopathological evaluation showed marked eosinophilic infiltration of the muscularis propria and serosa with concomitant mild acute inflammatory reaction (Figure 5). There was no evidence of malignancy, granuloma, TB, or parasites.

The constellation of clinical presentation and histopathological findings were suggestive of eosinophilic gastroenteritis. Subsequently, the patient was started on oral prednisone (20 mg/day). Two weeks later and with noticeable symptomatic improvement, the prednisone was tapered over a two-week period. After completion of steroids, the patient's abdominal pain and physical finding of ascites completely resolved and a peripheral blood count revealed an absolute eosinophil count of 300/*μ*l (nL < 450). Furthermore, IgE level dropped to 105 IU/mL and CT imaging of the abdominal and pelvis showed complete resolution of the ascites and small bowel thickening. Four months have elapsed since treatment and the patient remains asymptomatic on no medications.

## 3. Discussion

Eosinophilic gastroenteritis (EGE) is a rare condition characterized by recurrent [1] eosinophilic infiltration of portions of the gastrointestinal (GI) tract presenting with nonspecific GI symptoms in association with peripheral eosinophilia [2, 3]. The etiology and pathogenesis of EGE remain unclear. The role of allergy in recruitment of eosinophils to the GI tract remains controversial. Several studies have shown that half of the patients with EGE have preexisting history of atopy [4, 5]. In addition, serum IgE levels are elevated in some patients; yet rarely IgE antibodies are directed against identified food allergens. Furthermore, hypoallergenic diets have not been shown to be of significant benefit in treating EGE. Regardless of the initial trigger, activated tissue eosinophils release various chemoattractive cytokines resulting in recruitment of more eosinophils into the affected tissues [3, 6, 7].

Eosinophilic tissue infiltration can affect any of the three layers of the digestive apparatus with symptoms varying according to the affected layer. The most common classification of EGE based on the involved layer of the GI tract is known as Klein's classification. Subsequently, there are three subtypes of EGE (mucosal, muscular, and subserosal), with some degree of overlap [2, 4]. Data are insufficient in regard to the true prevalence of EGE and each of its subtypes. However, the mucosal form is the most common followed by muscular and lastly subserosal [6].

In a study of 15 patients with EGE, Chen et al. reported abdominal pain and diarrhea as the most common presenting symptoms. One-third of the patients had history of allergy and more than 80% were found to have peripheral eosinophilia. Histological examination revealed that 47% of the patients had mucosal type of EGE, 13% muscular, and 40% subserosal [8]. The prevalence of subserosal form of EGE varies among different studies. A clinicopathological study of 40 patients with EGE showed predominant mucosal disease in 57% patients, muscular (30%), and subserosal disease (13%) [4].

Symptoms of EGE are nonspecific and overlap with many other GI and systemic diseases. Mucosal subtype of EGE often presents with abdominal pain, nausea, vomiting, and/or diarrhea. Eosinophilic infiltration of the tunica muscularis results in a thickened rigid gut that produces symptoms of intestinal obstruction [6, 8]. Finally, patients with subserosal EGE have ascites as the source of their symptoms [6, 9–11]. Furthermore, this subgroup is clinically distinct in having abdominal bloating, higher eosinophil counts, and dramatic response to steroid therapy [4]. Eosinophilia is a distinguishing feature of ascites in patients with subserosal EGE. Further characterization of 42 patients with this subtype of EGE by Durieu et al. revealed female predominance with 75% of the patients being females 40 years and older. Moreover, the study showed that 69% of these patients had blood eosinophilia and 11% had pleural effusion. Eosinophilic infiltrations of the digestive tract were identified by tissue biopsy in 63% of the cases [5].

The diagnosis of EGE is established on high clinical suspicion in conjunction with suggestive histopathologic findings. Although peripheral eosinophilia is very common in all subtypes of EGE, it can be absent in as high as 23% of cases. In addition, 25% of the patients may have moderately elevated erythrocyte sedimentation rate (ESR) [4]. Thus, tissue samples are essential for confirming the diagnosis and classical findings include sheaths of eosinophils in the involved layer [7]. Nevertheless, this is not always an easy task as multiple endoscopic biopsies may be required due to the patchiness of the disease and diagnosis can be missed in up to 25% of cases [4]. Moreover, in cases where the diagnosis remains uncertain, CT imaging can help in localizing areas of thickened bowel suitable for surgical full thickness biopsy [7, 12, 13]. In contrast to the other two types, subserosal EGE may be confirmed by ascitic fluid analysis, which in the majority of cases reveals predominant eosinophilia reaching up to 99% of the white cells [14, 15]. Rarely, patients with EGE present with abdominal adenopathy resembling malignancy [16].

No test specific for EGE is available and prior to establishing such a diagnosis, a number of GI and systemic diseases should be excluded. Many of these disorders have similar presentations and may be associated with eosinophilia. This includes intestinal parasitic infections, malignancies such as lymphoma; gastric cancer; and colon cancer, inflammatory bowel disease, and more rarely systemic vasculitides such as polyarteritis nodosa and Churg-Strauss syndrome. The distinctive histologic features of EGE are absent in these diseases. Another major differential diagnosis of EGE is idiopathic hypereosinophilic syndrome, a condition associated with marked peripheral eosinophilia and gastroenteritis. In addition to possible involvement of the GI tract, this systemic entity may involve the heart, lungs, brain, and kidneys and frequently has a progressive course, while EGE lacks any extraintestinal manifestations [4, 6]. 

Available data on the natural history and therapy of EGE remains scarce. Untreated patients can remit spontaneously or progress to develop severe malabsorption. In most cases, the disease is essentially benign and pharmacologic therapy is not always indicated [1]. Many patients have been reported to spontaneously recover over a period of days [17]. In particular, the outcome of eosinophilic ascites was favorable in 90% and relapses occurred in 26% of 42 cases studied by Durieu et al. [5]. Nevertheless, due to insufficient understanding of the pathophysiology of EGE, treatment remains empiric and guided by the severity of symptoms. More symptomatic patients require therapy with prednisone (20 to 40 mg/day). A two-week course produces dramatic clinical improvement regardless of the histological subtype of EGE. Rapid tapering over another two weeks is sufficient to keep the majority of patients in remission. Furthermore, most patients have no recurrences, some relapse months to years after the prednisone taper yet respond to repeated short course of treatment, and only a minority requires long-term treatment with low-dose prednisone (5 to 10 mg/day). Others develop periodic flares after months or years. In addition to prednisone, small studies have described some success in using medications such as oral cromolyn, ketotifen, montelukast, and humanized anti-interleukin-5 antibody [6, 7, 18].

In the study by Chen et al., 13 out of 15 patients with EGE required treatment with prednisolone (10–40 mg/day) resulting in complete resolution of symptoms within two weeks. However, more than one-third of the treated patients relapsed in 12 months and 13% required long-term treatment with prednisolone (5 to 10 mg/day) [8]. In cases that fail to respond to corticosteroids, treatment with azathioprine or 6-mercaptopurine should be considered [19].

This case report reviews some of the characteristic clinical, laboratory, and histopathological findings of a rare, readily treatable, and easily missed disease. Due to the relatively nonspecific symptoms, this diagnosis should be considered in patients with ascites of unclear etiology, nonspecific bowel thickening by imaging studies and, otherwise, negative workup for parasitic infection and malignancy. Additionally, while peripheral blood or ascitic fluid eosinophilia is suggestive, its absence does not exclude the possibility of this diagnosis. Furthermore, prompt therapy with low-dose prednisone may reduce the duration and severity of symptoms. Thus, awareness of this condition and timely diagnosis and initiation of treatment would be of major importance.
